# (Benzoato-κ^2^ *O*,*O*′)(quinoline-2-carboxyl­ato-κ^2^ *N*,*O*)(quinoline-2-carboxylic acid-κ^2^ *N*,*O*)copper(II)

**DOI:** 10.1107/S1600536808014268

**Published:** 2008-05-21

**Authors:** Nuno D. Martins, Manuela Ramos Silva, Joana A. Silva, Ana Matos Beja, Abilio J. F.N. Sobral

**Affiliations:** a CEMDRX, Physics Department, University of Coimbra, P-3004-516 Coimbra, Portugal; b Chemistry Department, University of Coimbra, P-3004-516 Coimbra, Portugal

## Abstract

The crystal structure of the title compound, [Cu(C_10_H_6_NO_2_)(C_7_H_5_O_2_)(C_10_H_7_NO_2_)], contains copper(II) ions five-coordinated in a distorted trigonal-bipyramidal environment. The equatorial plane is occupied by three O atoms, one from the carboxyl­ate group of the benzoate ion considered as occupying a single coordination site, the other two from two carboxyl­ate groups of the quinaldic acid and quinaldate ligands. The axial positions are occupied by the N atoms of the quinoline ring system. The metal ion lies on a twofold axis that bisects the benzoate ion. The quinaldate and quinaldic acid ligands are equivalent by symmetry, and the carboxyl­ate/carboxyl groups are disordered. The disordered H atom is shared between the carboxyl­ate groups of adjacent quinaldic acid mol­ecules. Such hydrogen bonds delineate zigzag chains that run along the *c* axis. The structure is very similar to that of the Mn^II^ analog.

## Related literature

For related literature, see: Zurowska *et al.* (2007 ▶); Dobrzynska *et al.* (2005 ▶); Kumar & Gandotra (1980 ▶); Catterick *et al.* (1974 ▶); Martins *et al.* (2008 ▶).
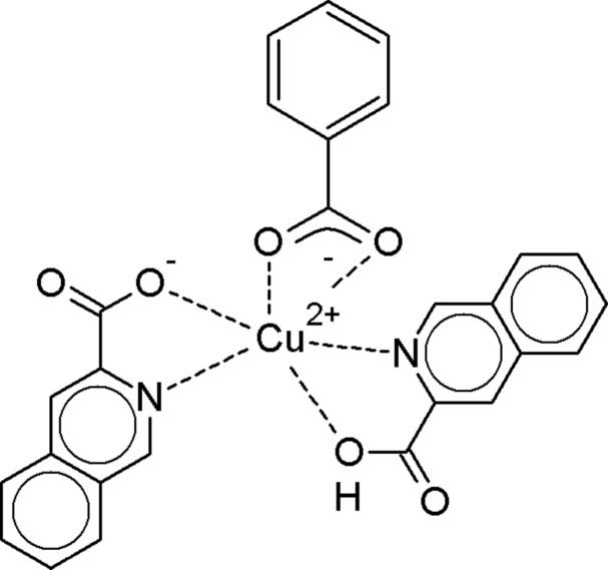


## Experimental

### 

#### Crystal data

[Cu(C_10_H_6_NO_2_)(C_7_H_5_O_2_)(C_10_H_7_NO_2_)]
*M* *_r_* = 529.97
Monoclinic, 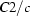

*a* = 19.1140 (9) Å
*b* = 11.4665 (5) Å
*c* = 12.1885 (8) Å
β = 118.788 (1)°
*V* = 2341.2 (2) Å^3^
*Z* = 4
Mo *K*α radiation
μ = 0.98 mm^−1^
*T* = 293 (2) K
0.26 × 0.22 × 0.20 mm

#### Data collection

Bruker APEX CCD area-detector diffractometer
Absorption correction: multi-scan (*SADABS*; Sheldrick, 2000 ▶) *T*_min_ = 0.75, *T*_max_ = 0.82
27106 measured reflections
2928 independent reflections
2536 reflections with *I* > 2σ(*I*)
*R*_int_ = 0.019

#### Refinement

*R*[*F* ^2^ > 2σ(*F* ^2^)] = 0.037
*wR*(*F* ^2^) = 0.104
*S* = 1.06
2928 reflections
178 parameters
H-atom parameters constrained
Δρ_max_ = 0.38 e Å^−3^
Δρ_min_ = −0.32 e Å^−3^

### 

Data collection: *SMART* (Bruker, 2003 ▶); cell refinement: *SAINT* (Bruker, 2003 ▶); data reduction: *SAINT*; program(s) used to solve structure: *SHELXS97* (Sheldrick, 2008 ▶); program(s) used to refine structure: *SHELXL97* (Sheldrick, 2008 ▶); molecular graphics: *ORTEPII* (Johnson, 1976 ▶); software used to prepare material for publication: *SHELXL97*.

## Supplementary Material

Crystal structure: contains datablocks global, I. DOI: 10.1107/S1600536808014268/bt2705sup1.cif

Structure factors: contains datablocks I. DOI: 10.1107/S1600536808014268/bt2705Isup2.hkl

Additional supplementary materials:  crystallographic information; 3D view; checkCIF report

## Figures and Tables

**Table 1:** Hydrogen-bond geometry (Å, °)

*D*—H⋯*A*	*D*—H	H⋯*A*	*D*⋯*A*	*D*—H⋯*A*
O3—H1⋯O4^i^	0.82	1.76	2.560 (3)	165

Symmetry code: (i) 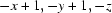.

**Fig. 1.**
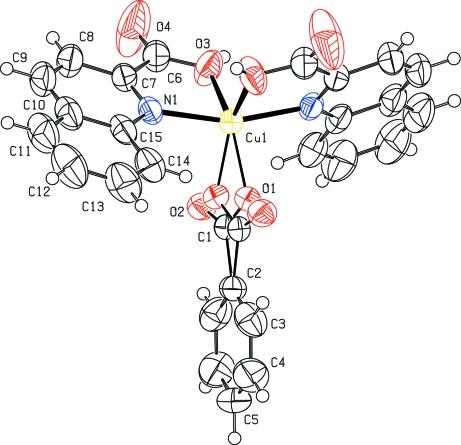
ORTEPII (Johnson, 1976) plot of the title compound. Displacement ellipsoids are drawn at the 50% level.

**Fig. 2.**
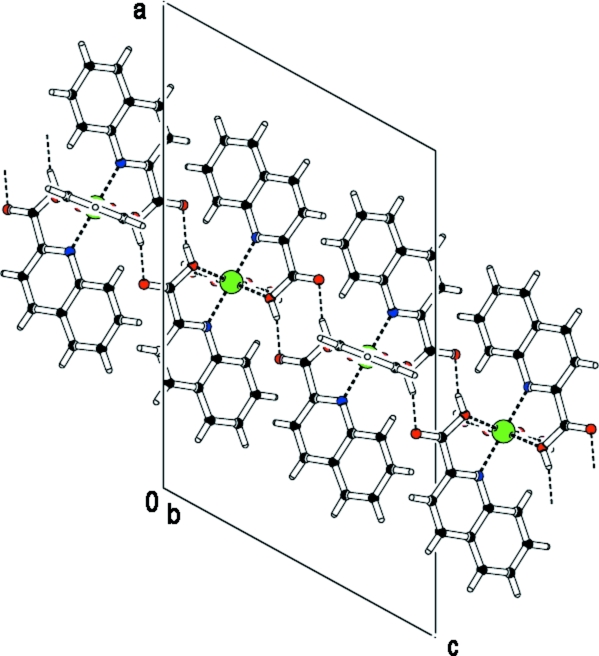
A chain formed via H-bonds running along the c axis.

## supplementary crystallographic information

### Comment

Some compounds with quinoline derivatives, transition metal ions and halide ions exhibit interesting magnetic properties related with the formation of low dimensional elements (Zurowska *et al.*, 2007; Dobrzynska *et al.*, 2005; Kumar & Gandotra, 1980; Catterick *et al.*, 1974). The crystal structure of the title compound, I, Cu (C_7_H_5_O_2_) (C_10_H_7_NO_2_)(C_10_H_6_NO_2_), consists of copper(II) ions five-coordinated in a distorted trigonal-bipyramidal environment (Fig. 1). The equatorial plane is occupied by three oxygen atoms with Cu—O distances of 1.970 (3) and 2.0553 (16) Å. One equatorial O atom belongs to the carboxylate group of the benzoate ion and each of the two quinoline molecules supply another O atom to the Cu coordination environment. The axial positions are occupied by the nitrogen atoms of the quinoline ring system, with distance 2.0424 (16) Å. There is a two fold axis running through the metal positions and almost longitudinally through the benzoate ion, that is thus disordered over two positions. The non-coordinating benzoate O atom is situated at a 2.678 (3)Å distance from the metal ion. With the exception of above mentioned benzoate disorder, the title compound shows a very similar arrangement to that of the Mn(II) analog (Martins *et al.*, 2008). The complexes are joined together by hydrogen bonds between the carboxylate/carboxyl groups of adjacent quinaldate/quinaldic acid molecules, forming zigzag chains that run along the *c* axis (Fig. 2). The shared hydrogen atom is disordered and the quinoline molecules are statistically neutral or negatively charged.

### Experimental

Approximately 0.12 mmol of 2-quinolinecarboxaldehyde (Sigma, 97%) was added to 0.12 mmol of copper chloride in an 10 ml dimethylformamide/benzoic acid solution. After a few weeks, single crystals of suitable quality were grown from the solution. The refined structure shows that the crystals incorporated a different quinoline derivative than that expected showing the material purchased from Sigma was contaminated.

### Refinement

H-atoms were positioned geometrically and refined using a riding model with C—H=0.93 Å, *U*_iso_(H)=1.2*U*_eq_(C). The carboxylic hydrogen atom, that could be located in a difference map, was positioned geometrically and refined within a riding model (HFIX 83), its occupancy was fixed to 0.5.

### Crystal data

**Table:**

[Cu(C_10_H_6_NO_2_)(C_7_H_5_O_2_)(C_10_H_7_NO_2_)]	*F*_000_ = 1084
*M**_r_* = 529.97	*D*_x_ = 1.504 Mg m^−^^3^
Monoclinic, *C*2/*c*	Mo *K*α radiation λ = 0.71073 Å
Hall symbol: -C 2yc	Cell parameters from 4050 reflections
*a* = 19.1140 (9) Å	θ = 3.2–26.8º
*b* = 11.4665 (5) Å	µ = 0.98 mm^−^^1^
*c* = 12.1885 (8) Å	*T* = 293 (2) K
β = 118.7880 (10)º	Block, green
*V* = 2341.2 (2) Å^3^	0.26 × 0.22 × 0.20 mm
*Z* = 4	

### Data collection

**Table:**

Bruker APEX CCD area-detector diffractometer	2928 independent reflections
Radiation source: fine-focus sealed tube	2536 reflections with *I* > 2σ(*I*)
Monochromator: graphite	*R*_int_ = 0.019
*T* = 293(2) K	θ_max_ = 28.4º
φ and ω scans	θ_min_ = 2.2º
Absorption correction: multi-scan(SADABS; Sheldrick, 2000)	*h* = −24→25
*T*_min_ = 0.75, *T*_max_ = 0.82	*k* = −15→15
27106 measured reflections	*l* = −16→16

### Refinement

**Table:**

Refinement on *F*^2^	Secondary atom site location: difference Fourier map
Least-squares matrix: full	Hydrogen site location: inferred from neighbouring sites
*R*[*F*^2^ > 2σ(*F*^2^)] = 0.037	H-atom parameters constrained
*wR*(*F*^2^) = 0.104	*w* = 1/[σ^2^(*F*_o_^2^) + (0.0492*P*)^2^ + 2.1952*P*] where *P* = (*F*_o_^2^ + 2*F*_c_^2^)/3
*S* = 1.06	(Δ/σ)_max_ < 0.001
2928 reflections	Δρ_max_ = 0.38 e Å^−^^3^
178 parameters	Δρ_min_ = −0.32 e Å^−^^3^
Primary atom site location: structure-invariant direct methods	Extinction correction: none

### Special details

**Table:**

Geometry. All e.s.d.'s (except the e.s.d. in the dihedral angle between two l.s. planes) are estimated using the full covariance matrix. The cell e.s.d.'s are taken into account individually in the estimation of e.s.d.'s in distances, angles and torsion angles; correlations between e.s.d.'s in cell parameters are only used when they are defined by crystal symmetry. An approximate (isotropic) treatment of cell e.s.d.'s is used for estimating e.s.d.'s involving l.s. planes.
Refinement. Refinement of *F*^2^ against ALL reflections. The weighted *R*-factor *wR* and goodness of fit *S* are based on *F*^2^, conventional *R*-factors *R* are based on *F*, with *F* set to zero for negative *F*^2^. The threshold expression of *F*^2^ > σ(*F*^2^) is used only for calculating *R*-factors(gt) *etc*. and is not relevant to the choice of reflections for refinement. *R*-factors based on *F*^2^ are statistically about twice as large as those based on *F*, and *R*- factors based on ALL data will be even larger.

### Fractional atomic coordinates and isotropic or equivalent isotropic displacement parameters (Å^2^)

**Table:**

	*x*	*y*	*z*	*U*_iso_*/*U*_eq_	Occ. (<1)
Cu1	0.5000	0.31494 (3)	0.2500	0.04408 (13)	
O1	0.51571 (18)	0.1520 (3)	0.3083 (3)	0.0467 (7)	0.50
O2	0.47650 (18)	0.1264 (3)	0.1065 (3)	0.0517 (7)	0.50
C1	0.4960 (2)	0.0874 (4)	0.2116 (3)	0.0406 (9)	0.50
O3	0.49011 (9)	0.42154 (18)	0.10765 (18)	0.0766 (6)	
H1	0.5218	0.4412	0.0837	0.115*	0.50
O4	0.39680 (14)	0.5024 (3)	−0.0713 (3)	0.1504 (15)	
N1	0.37963 (10)	0.34319 (16)	0.15664 (16)	0.0460 (4)	
C2	0.5000	−0.0419 (3)	0.2500	0.0568 (8)	
C3	0.50955 (16)	−0.1029 (3)	0.3531 (2)	0.0671 (7)	
H3	0.5165	−0.0628	0.4239	0.080*	
C4	0.50904 (19)	−0.2216 (3)	0.3528 (3)	0.0774 (8)	
H4	0.5149	−0.2620	0.4228	0.093*	
C5	0.5000	−0.2814 (3)	0.2500	0.0767 (11)	
H5	0.5000	−0.3625	0.2500	0.092*	
C6	0.41763 (13)	0.4477 (2)	0.0220 (2)	0.0563 (5)	
C7	0.35528 (12)	0.40982 (19)	0.05672 (19)	0.0492 (5)	
C8	0.27586 (13)	0.4476 (2)	−0.0157 (2)	0.0631 (6)	
H8	0.2609	0.4935	−0.0865	0.076*	
C9	0.22163 (14)	0.4154 (3)	0.0204 (3)	0.0690 (7)	
H9	0.1688	0.4392	−0.0260	0.083*	
C10	0.24486 (14)	0.3469 (2)	0.1267 (3)	0.0632 (6)	
C11	0.19152 (17)	0.3123 (3)	0.1708 (4)	0.0827 (9)	
H11	0.1385	0.3361	0.1280	0.099*	
C12	0.21650 (19)	0.2459 (4)	0.2729 (4)	0.0950 (11)	
H12	0.1807	0.2246	0.3007	0.114*	
C13	0.2958 (2)	0.2080 (3)	0.3386 (3)	0.0862 (9)	
H13	0.3122	0.1617	0.4095	0.103*	
C14	0.34976 (15)	0.2385 (3)	0.2997 (2)	0.0657 (6)	
H14	0.4022	0.2122	0.3431	0.079*	
C15	0.32525 (13)	0.30941 (19)	0.1942 (2)	0.0522 (5)	

### Atomic displacement parameters (Å^2^)

**Table:**

	*U*^11^	*U*^22^	*U*^33^	*U*^12^	*U*^13^	*U*^23^
Cu1	0.03721 (19)	0.03809 (19)	0.0498 (2)	0.000	0.01525 (15)	0.000
O1	0.0443 (16)	0.0374 (16)	0.0491 (16)	−0.0029 (13)	0.0152 (13)	−0.0017 (14)
O2	0.0571 (17)	0.0465 (17)	0.0476 (16)	−0.0006 (13)	0.0221 (14)	0.0053 (13)
C1	0.0313 (17)	0.039 (2)	0.047 (2)	0.0006 (17)	0.015 (2)	−0.0005 (15)
O3	0.0441 (8)	0.0868 (13)	0.0932 (13)	0.0102 (8)	0.0286 (8)	0.0495 (11)
O4	0.0687 (14)	0.217 (4)	0.144 (2)	0.0178 (17)	0.0337 (15)	0.124 (2)
N1	0.0362 (8)	0.0478 (9)	0.0496 (9)	−0.0066 (7)	0.0172 (7)	−0.0082 (7)
C2	0.0363 (13)	0.0389 (15)	0.094 (2)	0.000	0.0301 (15)	0.000
C3	0.0658 (15)	0.0756 (17)	0.0649 (14)	−0.0121 (13)	0.0356 (12)	−0.0229 (13)
C4	0.087 (2)	0.0765 (18)	0.0623 (15)	−0.0115 (15)	0.0309 (14)	0.0163 (14)
C5	0.080 (3)	0.0383 (16)	0.092 (3)	0.000	0.026 (2)	0.000
C6	0.0461 (11)	0.0635 (14)	0.0533 (11)	−0.0014 (10)	0.0192 (9)	0.0046 (10)
C7	0.0401 (10)	0.0490 (11)	0.0510 (11)	−0.0015 (8)	0.0158 (8)	−0.0064 (9)
C8	0.0441 (11)	0.0684 (15)	0.0636 (13)	0.0069 (10)	0.0155 (10)	−0.0013 (12)
C9	0.0395 (11)	0.0742 (17)	0.0812 (17)	0.0045 (11)	0.0194 (11)	−0.0122 (14)
C10	0.0425 (11)	0.0688 (15)	0.0784 (16)	−0.0107 (10)	0.0291 (11)	−0.0234 (13)
C11	0.0506 (14)	0.101 (2)	0.105 (2)	−0.0152 (14)	0.0443 (16)	−0.0185 (19)
C12	0.0675 (18)	0.126 (3)	0.114 (3)	−0.0322 (19)	0.0611 (19)	−0.022 (2)
C13	0.0775 (19)	0.106 (3)	0.088 (2)	−0.0260 (17)	0.0494 (17)	0.0001 (17)
C14	0.0536 (13)	0.0770 (17)	0.0695 (14)	−0.0168 (12)	0.0320 (12)	−0.0058 (13)
C15	0.0416 (10)	0.0560 (13)	0.0595 (12)	−0.0126 (9)	0.0247 (9)	−0.0154 (10)

### Geometric parameters (Å, °)

**Table:**

Cu1—O1^i^	1.970 (3)	C3—C4	1.361 (4)
Cu1—O1	1.970 (3)	C3—H3	0.9300
Cu1—N1	2.0424 (16)	C4—C5	1.364 (4)
Cu1—N1^i^	2.0424 (16)	C4—H4	0.9300
Cu1—O3^i^	2.0553 (16)	C5—C4^i^	1.364 (4)
Cu1—O3	2.0553 (16)	C5—H5	0.9300
O1—C1^i^	0.777 (4)	C6—C7	1.508 (3)
O1—O2^i^	1.018 (4)	C7—C8	1.407 (3)
O1—O1^i^	1.249 (7)	C8—C9	1.358 (4)
O1—C1	1.285 (4)	C8—H8	0.9300
O2—O1^i^	1.018 (4)	C9—C10	1.392 (4)
O2—C1	1.233 (5)	C9—H9	0.9300
C1—O1^i^	0.777 (4)	C10—C15	1.416 (3)
C1—C1^i^	0.872 (8)	C10—C11	1.419 (4)
C1—C2	1.545 (5)	C11—C12	1.336 (5)
O3—C6	1.306 (3)	C11—H11	0.9300
O3—H1	0.8200	C12—C13	1.400 (5)
O4—C6	1.187 (3)	C12—H12	0.9300
N1—C7	1.318 (3)	C13—C14	1.371 (4)
N1—C15	1.378 (3)	C13—H13	0.9300
C2—C3^i^	1.372 (3)	C14—C15	1.398 (4)
C2—C3	1.372 (3)	C14—H14	0.9300
C2—C1^i^	1.545 (5)		
			
O1^i^—Cu1—O1	36.95 (19)	C3—C2—C1^i^	104.3 (2)
O1^i^—Cu1—N1	90.82 (10)	C4—C3—C2	120.7 (2)
O1—Cu1—N1	106.65 (10)	C4—C3—H3	119.6
O1^i^—Cu1—N1^i^	106.65 (10)	C2—C3—H3	119.6
O1—Cu1—N1^i^	90.82 (10)	C3—C4—C5	120.2 (3)
N1—Cu1—N1^i^	161.74 (10)	C3—C4—H4	119.9
O1^i^—Cu1—O3^i^	143.58 (12)	C5—C4—H4	119.9
O1—Cu1—O3^i^	108.87 (12)	C4^i^—C5—C4	119.6 (4)
N1—Cu1—O3^i^	89.83 (7)	C4^i^—C5—H5	120.2
N1^i^—Cu1—O3^i^	79.30 (7)	C4—C5—H5	120.2
O1^i^—Cu1—O3	108.87 (12)	O4—C6—O3	128.6 (2)
O1—Cu1—O3	143.58 (12)	O4—C6—C7	118.4 (2)
N1—Cu1—O3	79.30 (7)	O3—C6—C7	112.78 (19)
N1^i^—Cu1—O3	89.83 (7)	N1—C7—C8	123.5 (2)
O3^i^—Cu1—O3	107.02 (13)	N1—C7—C6	116.81 (18)
C1^i^—O1—O2^i^	85.6 (5)	C8—C7—C6	119.6 (2)
O2^i^—O1—O1^i^	156.1 (4)	C9—C8—C7	118.3 (2)
O2^i^—O1—C1	127.1 (3)	C9—C8—H8	120.9
C1^i^—O1—Cu1	145.0 (5)	C7—C8—H8	120.9
O2^i^—O1—Cu1	124.3 (3)	C8—C9—C10	120.3 (2)
O1^i^—O1—Cu1	71.52 (10)	C8—C9—H9	119.9
C1—O1—Cu1	106.8 (3)	C10—C9—H9	119.9
O1^i^—C1—C1^i^	102.2 (5)	C9—C10—C15	118.9 (2)
O1^i^—C1—O2	55.5 (4)	C9—C10—C11	123.0 (3)
C1^i^—C1—O2	157.5 (4)	C15—C10—C11	118.1 (3)
O1^i^—C1—O1	69.6 (5)	C12—C11—C10	120.8 (3)
O2—C1—O1	123.6 (4)	C12—C11—H11	119.6
O1^i^—C1—C2	166.6 (5)	C10—C11—H11	119.6
C1^i^—C1—C2	73.61 (15)	C11—C12—C13	120.8 (3)
O2—C1—C2	127.5 (4)	C11—C12—H12	119.6
O1—C1—C2	109.0 (3)	C13—C12—H12	119.6
C6—O3—Cu1	116.24 (14)	C14—C13—C12	120.7 (3)
C6—O3—H1	109.5	C14—C13—H13	119.7
Cu1—O3—H1	132.9	C12—C13—H13	119.7
C7—N1—C15	118.85 (18)	C13—C14—C15	119.5 (3)
C7—N1—Cu1	114.11 (14)	C13—C14—H14	120.3
C15—N1—Cu1	126.73 (15)	C15—C14—H14	120.3
C3^i^—C2—C3	118.6 (3)	N1—C15—C14	119.8 (2)
C3^i^—C2—C1	104.3 (2)	N1—C15—C10	120.2 (2)
C3—C2—C1	137.1 (2)	C14—C15—C10	120.0 (2)
C3^i^—C2—C1^i^	137.1 (2)		
			
O1^i^—Cu1—O1—C1^i^	−15.7 (7)	O3^i^—Cu1—N1—C15	−65.38 (18)
N1—Cu1—O1—C1^i^	52.4 (9)	O3—Cu1—N1—C15	−172.74 (19)
N1^i^—Cu1—O1—C1^i^	−132.9 (8)	O1^i^—C1—C2—C3^i^	108 (2)
O3^i^—Cu1—O1—C1^i^	148.1 (8)	C1^i^—C1—C2—C3^i^	−179.0 (5)
O3—Cu1—O1—C1^i^	−42.1 (9)	O2—C1—C2—C3^i^	9.9 (5)
O1^i^—Cu1—O1—O2^i^	−159.9 (6)	O1—C1—C2—C3^i^	−170.9 (3)
N1—Cu1—O1—O2^i^	−91.8 (4)	O1^i^—C1—C2—C3	−72 (2)
N1^i^—Cu1—O1—O2^i^	82.8 (4)	C1^i^—C1—C2—C3	1.4 (7)
O3^i^—Cu1—O1—O2^i^	3.9 (4)	O2—C1—C2—C3	−169.7 (3)
O3—Cu1—O1—O2^i^	173.7 (3)	O1—C1—C2—C3	9.5 (4)
N1—Cu1—O1—O1^i^	68.1 (3)	O1^i^—C1—C2—C1^i^	−73 (2)
N1^i^—Cu1—O1—O1^i^	−117.2 (3)	O2—C1—C2—C1^i^	−171.1 (9)
O3^i^—Cu1—O1—O1^i^	163.8 (3)	O1—C1—C2—C1^i^	8.2 (3)
O3—Cu1—O1—O1^i^	−26.4 (4)	C3^i^—C2—C3—C4	−0.4 (2)
O1^i^—Cu1—O1—C1	5.6 (2)	C1—C2—C3—C4	179.1 (3)
N1—Cu1—O1—C1	73.7 (3)	C1^i^—C2—C3—C4	179.9 (3)
N1^i^—Cu1—O1—C1	−111.6 (2)	C2—C3—C4—C5	0.9 (4)
O3^i^—Cu1—O1—C1	169.4 (2)	C3—C4—C5—C4^i^	−0.4 (2)
O3—Cu1—O1—C1	−20.8 (3)	Cu1—O3—C6—O4	−176.0 (3)
O1^i^—O2—C1—C1^i^	6.7 (16)	Cu1—O3—C6—C7	9.5 (3)
O1^i^—O2—C1—O1	−15.3 (5)	C15—N1—C7—C8	−0.9 (3)
O1^i^—O2—C1—C2	163.8 (7)	Cu1—N1—C7—C8	−175.00 (18)
C1^i^—O1—C1—O1^i^	152.6 (11)	C15—N1—C7—C6	177.99 (18)
O2^i^—O1—C1—O1^i^	155.8 (7)	Cu1—N1—C7—C6	3.9 (2)
Cu1—O1—C1—O1^i^	−9.2 (4)	O4—C6—C7—N1	175.9 (3)
O2^i^—O1—C1—C1^i^	3.3 (8)	O3—C6—C7—N1	−9.0 (3)
O1^i^—O1—C1—C1^i^	−152.6 (11)	O4—C6—C7—C8	−5.2 (4)
Cu1—O1—C1—C1^i^	−161.7 (7)	O3—C6—C7—C8	170.0 (2)
C1^i^—O1—C1—O2	166.0 (10)	N1—C7—C8—C9	1.2 (4)
O2^i^—O1—C1—O2	169.3 (3)	C6—C7—C8—C9	−177.7 (2)
O1^i^—O1—C1—O2	13.4 (4)	C7—C8—C9—C10	0.1 (4)
Cu1—O1—C1—O2	4.2 (5)	C8—C9—C10—C15	−1.5 (4)
C1^i^—O1—C1—C2	−13.3 (5)	C8—C9—C10—C11	178.7 (3)
O2^i^—O1—C1—C2	−10.0 (5)	C9—C10—C11—C12	179.6 (3)
O1^i^—O1—C1—C2	−165.9 (6)	C15—C10—C11—C12	−0.2 (4)
Cu1—O1—C1—C2	−175.0 (2)	C10—C11—C12—C13	−0.5 (6)
O1^i^—Cu1—O3—C6	81.1 (2)	C11—C12—C13—C14	0.1 (6)
O1—Cu1—O3—C6	97.5 (2)	C12—C13—C14—C15	0.9 (5)
N1—Cu1—O3—C6	−6.11 (19)	C7—N1—C15—C14	179.8 (2)
N1^i^—Cu1—O3—C6	−171.4 (2)	Cu1—N1—C15—C14	−6.9 (3)
O3^i^—Cu1—O3—C6	−92.6 (2)	C7—N1—C15—C10	−0.6 (3)
O1^i^—Cu1—N1—C7	−108.25 (18)	Cu1—N1—C15—C10	172.68 (16)
O1—Cu1—N1—C7	−142.16 (17)	C13—C14—C15—N1	178.1 (2)
N1^i^—Cu1—N1—C7	55.17 (14)	C13—C14—C15—C10	−1.5 (4)
O3^i^—Cu1—N1—C7	108.16 (16)	C9—C10—C15—N1	1.8 (3)
O3—Cu1—N1—C7	0.80 (15)	C11—C10—C15—N1	−178.4 (2)
O1^i^—Cu1—N1—C15	78.21 (19)	C9—C10—C15—C14	−178.6 (2)
O1—Cu1—N1—C15	44.3 (2)	C11—C10—C15—C14	1.2 (4)
N1^i^—Cu1—N1—C15	−118.37 (17)		

Symmetry codes: (i) −*x*+1, *y*, −*z*+1/2.

### Hydrogen-bond geometry (Å, °)

**Table:**

*D*—H···*A*	*D*—H	H···*A*	*D*···*A*	*D*—H···*A*
O3—H1···O4^ii^	0.82	1.76	2.560 (3)	165

Symmetry codes: (ii) −*x*+1, −*y*+1, −*z*.
